# Working-Boys' Club

**Published:** 1920-09-11

**Authors:** 


### Working-Boys' Club.

### AN APPEAL FOR HELP.

We have received a strong appeal from an influential committee on behalf of the Federation of London Working-Boys' Clubs. They state that it is intended to increase the number of boys' clubs in London in the hope that every boy on leaving school may have the chance of joining some club or similar organisation, where he can spend his time to greater advantage than in his overcrowded home or in the streets. The Federation represents at present over 100 boys' clubs, large and small, in all parts of greater London, and, with the prospect of a vastly larger number, it becomes essential to put the Federation into such a position that it will be capable of co-ordinating the work all over London, assisting in case of need those clubs which are unable to help themselves, and giving new clubs and their managers the benefits of its thirty years' experience.

The policy of the Federation has been to stimulate that spirit of loyalty to a team or club, learnt unconsciously by means of sports and games, which serves also to teach a boy to recognise and perform his duty to the State in time of need. How deep a sense of duty existed among the members of these boys' clubs was shown during the first year of the war, when nearly every member, on reaching military age, voluntarily enlisted, and boys of fifteen and sixteen clamoured to be allowed to do the same. The Federation has never previously made a public appeal, it has no funds, and the income of £150, subscribed by a few friends, is totally inadequate to carry on the work. It requires £1,000 a year to enable it to assist clubs which have no support such as is accorded to those backed by the public schools or colleges, to defray the cost of competitions, and to pay a good secretary and office expenses. In making the appeal, which is signed by Lord Desborough as president, the committee wish to make it clear that the clubs are not run as charities, but each member to the best of his ability pays his way. It is, however, essential that there should be some central authority, and that is why the Federation exists. But it can only do so if it is supported by voluntary contributions. Subscriptions should be sent to the Hon. Treasurer, 41 Memorial Hall Buildings, Farringdon Street, E.C. 4.

